# Monocytes and macrophages, implications for breast cancer migration and stem cell-like activity and treatment

**DOI:** 10.18632/oncotarget.4189

**Published:** 2015-05-19

**Authors:** Rebecca Ward, Andrew H. Sims, Alexander Lee, Christina Lo, Luke Wynne, Humza Yusuf, Hannah Gregson, Michael P. Lisanti, Federica Sotgia, Göran Landberg, Rebecca Lamb

**Affiliations:** ^1^ Breakthrough Breast Cancer Unit, Institute of Cancer Sciences, Paterson Institute for Cancer Research, University of Manchester, Manchester, UK; ^2^ Applied Bioinformatics of Cancer, University of Edinburgh Cancer Research Centre, Carrington Crescent, Edinburgh, UK; ^3^ Sahlgrenska Cancer Center, University of Gothenburg, Sweden

**Keywords:** macrophage, breast, stem-cells, migration

## Abstract

Macrophages are a major cellular constituent of the tumour stroma and contribute to breast cancer prognosis. The precise role and treatment strategies to target macrophages remain elusive. As macrophage infiltration is associated with poor prognosis and high grade tumours we used the THP-1 cell line to model monocyte-macrophage differentiation in co-culture with four breast cancer cell lines (MCF7, T47D, MDA-MB-231, MDA-MB-468) to model *in vivo* cellular interactions. Polarisation into M1 and M2 subtypes was confirmed by specific cell marker expression of ROS and HLA-DR, respectively. Co-culture with all types of macrophage increased migration of ER-positive breast cancer cell lines, while M2-macrophages increased mammosphere formation, compared to M1-macrophages, in all breast cancer cells lines. Treatment of cells with Zoledronate in co-culture reduced the “pro-tumourigenic” effects (increased mammospheres/migration) exerted by macrophages. Direct treatment of breast cancer cells in homotypic culture was unable to reduce migration or mammosphere formation.

Macrophages promote “pro-tumourigenic” cellular characteristics of breast cancer cell migration and stem cell activity. Zoledronate targets macrophages within the microenvironment which in turn, reduces the “pro-tumourigenic” characteristics of breast cancer cells. Zoledronate offers an exciting new treatment strategy for both primary and metastatic breast cancer.

## INTRODUCTION

Breast cancer is the most common disease in women in the Western world with an incidence rate of 600,000 and a mortality rate of 200,000 each year [1]. Breast cancer diagnosis and treatment has significantly improved in recent years with increased disease-free survival in breast cancer patients. Despite these improvements, a significant proportion of breast cancers are either resistant to treatment or show disease recurrence [2, 3]. It is essential that we improve our understanding of cancer biology and the complex interplay between the different cell types driving cancer, in order to develop more targeted therapies.

Loss of control of proliferation and resistance to apoptosis/necrosis is considered a hallmark of many cancer types including breast cancer [4]. Subsequent to primary tumour formation, metastasis may occur whereby cells acquire migratory capacity, invade into the surrounding tissue, enter the blood stream and lymphatic system and then extravasate and colonise a secondary site [5]. Recurrences at metastatic sites represent a major cause of mortality in breast cancer patients [6, 7]. Resistance to therapy impedes the improvement of breast cancer mortality rates within the clinic. Research suggests that cancer stem cells (CSCs) play an important role in breast cancer and therapy resistance [8] emphasising the need for new treatment strategies [9, 10]. Additionally, CSCs may be responsible for the initiation and regeneration of tumours [11, 12] and given their quiescent nature may be responsible for lasting residual disease and underlying tumour dormancy [8]. CSCs can be identified by various cellular markers including CD44^+^/CD24^−^ and ALDH1 expression, or by their ability to form mammospheres [13-15].

Breast cancer is a solid tumour therefore its surrounding microenvironment is particularly important. In breast cancer stroma, macrophages can occupy more than fifty percent of the breast tumour mass with studies showing a correlation between macrophage density and poor patient prognosis [16-19]. Macrophages are heterogeneous in population and can be classified as M1 or M2, polarising to each dependent on the stimuli present at time of activation [20, 21]. In tumours it has been suggested that M2 macrophages are most prevalent and that they directly promote breast cancer growth by secreting breast tumour mitogens and angiogenic factors [22-24] and are associated with poor prognosis in breast cancer patients [16, 25].

There is clear evidence that macrophages are a key component of the tumour stroma and influence breast cancer prognosis, it is therefore essential we understand the exact mechanisms and develop new treatments to target the microenvironment. One promising treatment is Zoledronate (zoledronic acid) a bisphosphonate used in the treatment of osteoporosis and advanced breast cancer due to its positive actions within bone. However it was observed that Zoledronate may also inhibit breast cancer progression and invasion [26] with recent data suggesting that which recent data suggests these actions may be via M2 pro-tumourigenic macrophages rather than directly on breast cancer cells [27].

Our study uses co-culture approaches to provide further insight into the mechanisms of tumour-associated macrophages by examining the influence of macrophages upon breast cancer cell proliferation, apoptosis, migration and stem-cell activity. Crucially, we demonstrate that Zoledronate treatment can overcome the “pro-tumourigenic” effects that macrophages exert within the microenvironment by reducing breast cancer migration and mammosphere formation.

## RESULTS

### Macrophage infiltration in primary breast tumours

**Figure 1:**
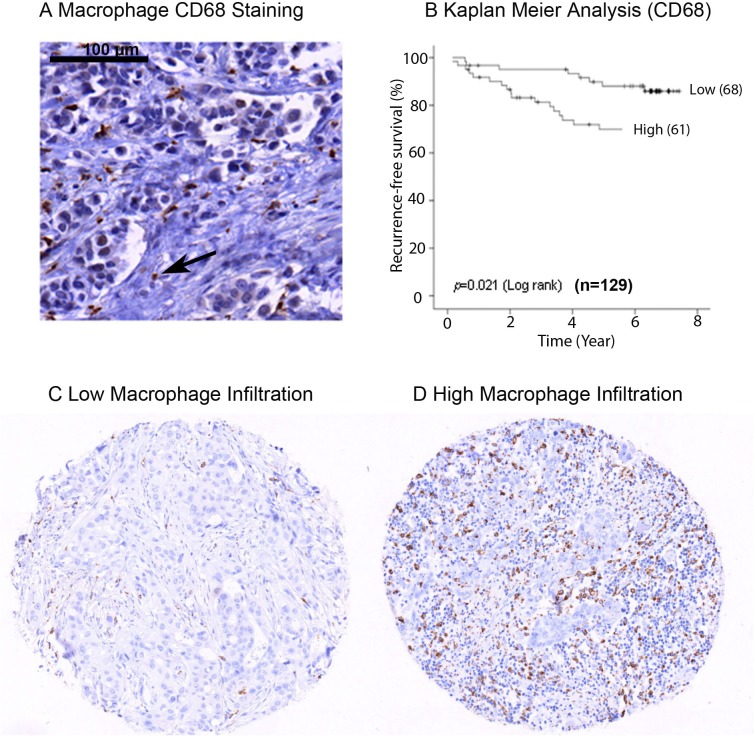
Macrophage infiltration in primary breast tumours IHC staining of formalin fixed breast cancer samples for CD68 **A.** Image showing CD68 expression marking the macrophage cell type **B.** Kaplan Meier analysis of CD68 expression grouped into high and low expression. High macrophage infiltration predicts decreased recurrence free survival **C.** Representative TMA core of breast tumours showing low macrophage infiltration **D.** Representative TMA core of breast tumours showing high macrophage infiltration.

**Figure 2:**
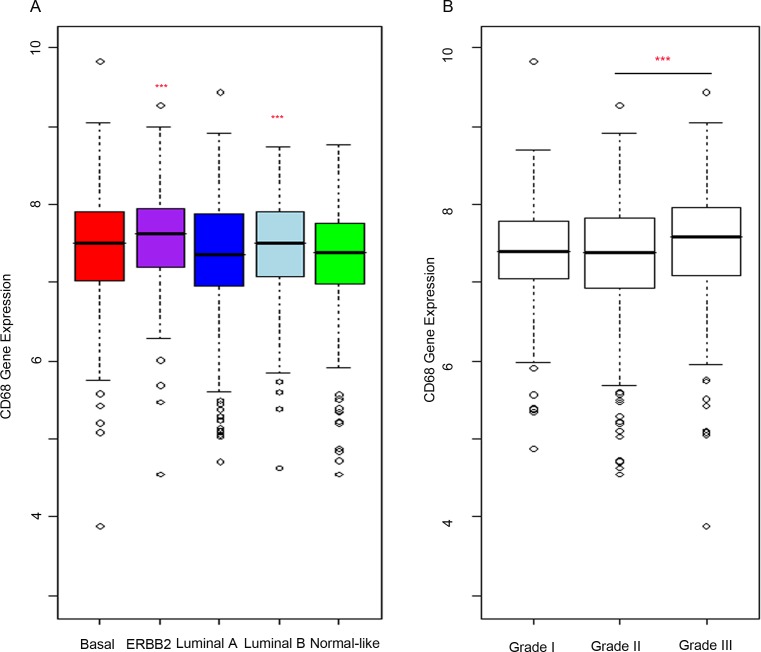
CD68 is more highly expressed in high grade breast tumours of ERRB2 and Luminal B subtypes mRNA analysis of breast cancer samples. CD68 gene expression (“203507_at” probeset, batch corrected data from six published studies) was used as a marker of macrophages and expression analysed within the breast cancer samples. **A.** Expression levels were compared in breast cancer subtypes (ERBB2, Basal, Luminal A, Luminal B) to that in normal-like samples. Macrophage infiltration is increased in Luminal A and ERBB2 subtypes compared to normal-like. **B.** Analysis of CD68 expression to grade showed significant positive correlation.

To confirm that macrophage infiltration is associated with poor prognosis in primary breast cancer, we analysed the expression of CD68 protein within a tissue microarray (TMA) containing formalin fixed tumours from 129 breast cancer patients (Figure 1A). High expression of CD68 and therefore high macrophage infiltration was predictive of poor recurrence free survival (*p* = 0.02) (Figure 1B). For analysis, expression of CD68 was divided into two groups, ‘low’ (Figure 1C) and ‘high’ (Figure 1D) relative to mean expression. Additionally, high macrophage infiltration was associated with more aggressive tumours, represented by high grade tumours (*p* = 0.02) and lymph node involvement (*p* = 0.04) (Table 1), but was not associated with ER or HER2 status. We also assessed CD68 mRNA expression in a cohort of 1107 breast cancer tumour tissue samples (containing tumour stroma) comprising six publically available gene expression data sets [28]. Increased expression was observed in both Luminal B and ERBB2 expressing tumours compared to normal-like breast tissue (Figure 2A). As with the protein data, grade 3 tumours showed significantly higher expression of the macrophage marker CD68 (Figure 2B).

**Table 1:** Clinico-pathological characteristics of breast cancer patients in relation to macrophage count (CD68 expression) Distributions of tumour macrophage number (CD68 expression) categorisations according to clinical-pathological and molecular parameters (percentages in parenthesis). Macrophage count was divided into low and high by the median value. High level of macrophage infiltration was associated with higher tumour grade and lymph node metastasis.

	Macrophage (CD68) n=129		p-value
	Low	High	
Number of patients	68 (52.7%)%)	61 (47.3%)	
Age (years)			
median (range)	66.5 (35-91)	64.0 (34-97)	0.854^1^
Tumour size			
≤20mm	39 (59.1%)	27 (40.9%)	0.160^3^
>20mm	29 (46.0%)	34 (54.0%)	
Grade			
I	11 (57.9%)	8 (42.1%)	0.023^2^
II	38 (63.3%)	22 (36.7%)	
III	19 (38.0%)	31 (62.0%)	
Lymph node status			
negative	41 (61.2%)	26 (38.8%)	0.044^3^
positive	22(42.3%)	30(57.7%)	
Oestrogen receptor status			
negative	5 (31.2%)	11 (68.8%)	0.106^3^
positive	63 (55.8%)	50 (44.2%)	
Progesterone receptor status			
negative	18 (46.2%)	21 (53.8%)	0.344^3^
positive	50 (55.6%)	40 (44.4%)	
HER2 status			
negative	62 (53.0%)	55 (47.0%)	0.735^3^
positive	4 (44.4%)	5 (55.6%)	
Ki67			
≤10%	1 (16.7%)	5 (83.3%)	1.000^2^
11-25%	27 (57.4%)	20 (42.6%)	
>25%	28 (48.3%)	30 (51.7%)	

1 Kruskel Wallis

2 Spearman's Rho

3 Fisher's Exact.

### Generating differentiated macrophages

**Figure 3:**
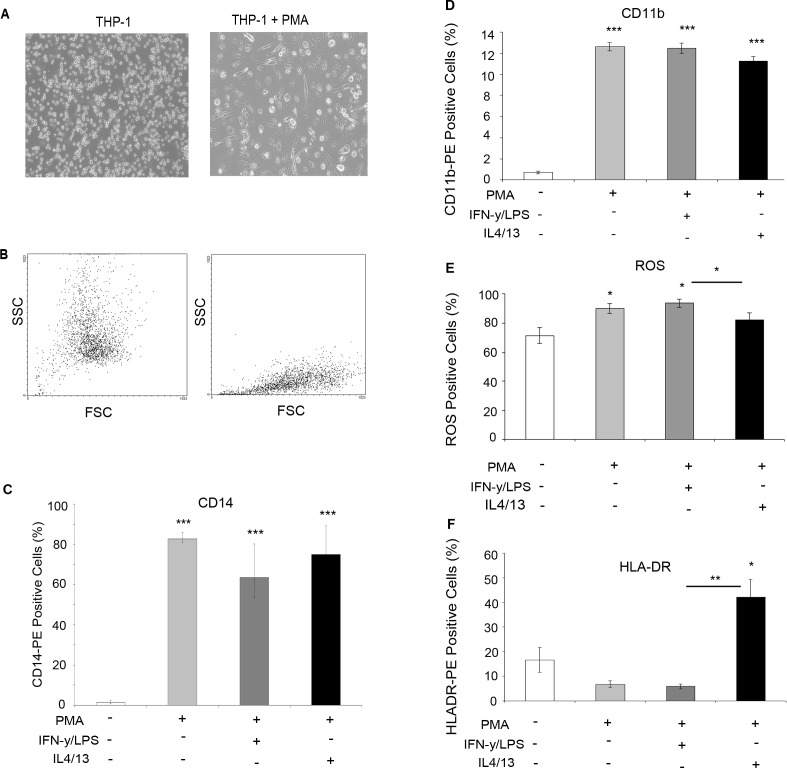
Differentiation of THP-1 monocytes into macrophages THP-1 monocytes were treated for three days with PMA to induce macrophage differentiation **A.** Bright-field imaging shows increased adherence **B.** FACS analysis of SSC and FSC confirmed altered morphology following addition of PMA (right panel). Cells were further treated with IFN /LPS (M1) or IL4/IL13 to achieve macrophage polarisation. FACS analysis showed increased **C.** CD14 expression and **D**. CD11b in macrophages vs THP-1 monocytes **E.** FACS analysis of ROS activity showed increased expression in THP-1 cells? treated with IFN/LPS **F.** FACS analysis of HLA-DR expression was increased in cells treated with IL4/IL13. Bar charts represent the mean % of cells, ±SEM. *P* values were generated using a two sided t-test assuming equal variance compared to either THP-1 monocytes, or between IFN/LPS and IL4/IL13 treated cells * indicates significance, *p* < 0.05. **p < 0.01 ***p < 0.001.

The THP-1 monocytic cell line was used as a model to generate differentiated macrophages for co-culture experiments. Phorbol myristate acetate (PMA) treatment induced differentiation measured by an increase in adherence (Figure 3A) and changes in morphology, indicated by altered forward scatter (FSC) and side scatter (SCC) FACS plots (Figure 3B). Flow cytometry analysis showed that macrophages expressed significantly increased levels of CD14 (Figure 3C) and CD11b (Figure 3D) confirming macrophage differentiation. In addition, lysotracker was used to confirm THP-1 monocyte to macrophage differentiation, with differentiated macrophages showing increased lysosome accumulation (Supplementary Figure 1). Further treatment of macrophages with LPS/IFN or IL4/IL13 induced polarisation into M1 and M2 subtypes marked by increased ROS production (Figure 3E) and HLA-DR (Figure 3F) expression, respectively.

### Monocytes and macrophages have no major effects on breast cancer cell growth, apoptosis or necrosis

**Figure 4:**
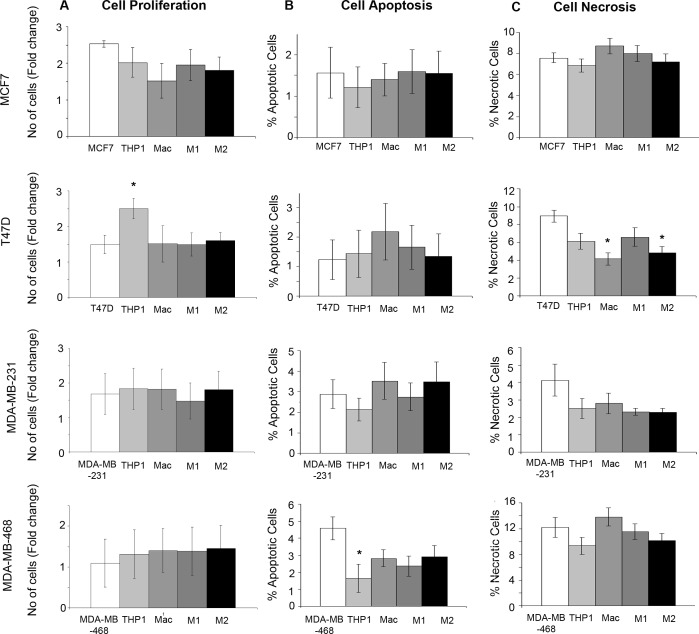
Effects of monocytes and macrophages on breast cancer growth, apoptosis and necrosis Breast cancer cell lines (MCF7, T47D, MDA-MB-231 and MDA-MB-468) were co-cultured with THP1-monocytes, macrophages or M1/M2 macrophages and compared to breast cancer cells alone. **A.** Proliferation was assessed by Alamar blue staining, **B.** apoptosis by Annexin staining and FACS analysis **C.** and necrosis by Propidium Ioidine staining and FACS analysis. Bar charts represent the mean % of cells, ±SEM. *P* values were generated using a two sided t-test assuming equal variance compared to breast cancer cells alone. * indicates significance, *p* < 0.05. ** *p* < 0.01 ***p < 0.001.

We co-cultured two ER+ and two ER− breast cancer cell lines, (MCF7, T47D, MDA-MB-231, MDA-MB-468), with THP-1 monocytes, macrophages and M1/M2 subtypes for 48hrs (Supplementary Figure 2). The only significant change in proliferation observed was with the co-culture of THP-1 monocytes and T47D cells where an increase in proliferation was observed (Figure 4A). Cell apoptosis determined by Annexin V staining was only significantly changed with the co-culture of THP-1 monocytes with MDA-MB-468 cells, where a reduction in apoptosis was seen (Figure 4B). Analysis of necrosis marked by Propidium Iodide staining showed a reduction in necrosis with co-culture of macrophages and M2 macrophages with T47D cells (Figure 4C). Overall, no consistent changes or patterns following co-culture were observed across the breast cancer cell lines.

### Monocytes and macrophages affect migration dependent upon breast cancer subtype

**Figure 5:**
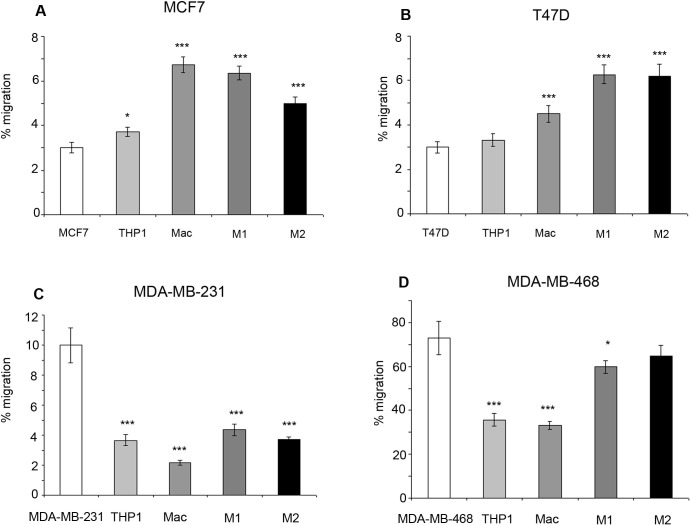
Effects of monocytes and macrophages on cell migration Breast cancer cell lines **a**) MCF7, **b**) T47D, **c**) MDAMB-231 and **d**) MDA-MB-468 were co-cultured with THP1-monocytes, macrophages or M1/M2 macrophages and compared to breast cancer cells alone. Migration of breast cancer cells was assessed by transwell inserts. Bar charts represent the mean % of cells, ±SEM. P values were generated using a two sided t-test assuming equal variance compared to breast cancer cells alone, or between cells co-cultured with M1 and M2 macrophages * indicates significance, p < 0.05. ** p < 0.01 ***p < 0.001.

Within ER+ breast cancer cell lines, co-culture with all types of macrophages including M1 and M2 subtypes increased breast cancer migration compared to breast cancer cell lines alone, or in co-culture with THP-1 monocytes. A small increase in migration was observed with co-culture of THP-1 monocytes and MCF7 cells (Figure 5A/5B). In comparison, co-culture of ER− breast cancer cells with THP-1 monoctyes or macrophages caused a decrease in migration compared to breast cancer cells alone. Interestingly, M1 and M2 subtypes caused increased migration in ER− breast cancer cells compared to co-culture with general macrophages (Figure 5C/5D).

### M2 macrophages increase mammosphere formation in breast cancer cell lines

**Figure 6:**
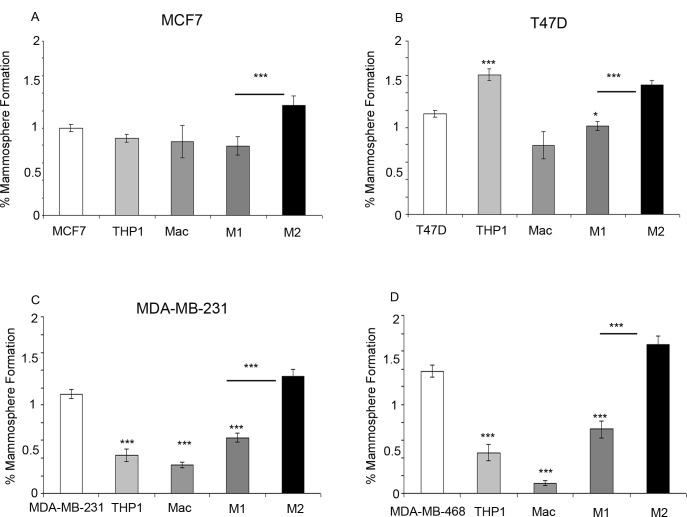
Effects of monocytes and macrophages on mammosphere formation Breast cancer cell lines **a**) MCF7, **b**) T47D, **c**) MDA-MB-231 and **d**) MDA-MB-468 were co-cultured with THP1-monocytes, macrophages or M1/M2 macrophages and compared to breast cancer cells alone. Mammosphere formation of breast cancer cells was assessed. Bar charts represent the mean % of cells, ±SEM. P values were generated using a two sided t-test assuming equal variance compared to breast cancer cells alone, or between cells co-cultured with M1 and M2 macrophages * indicates significance, p < 0.05. ** p < 0.01 ***p < 0.001.

Within ER+ breast cancer cell lines, co-culture with M2 macrophages significantly increased mammosphere formation compared to co-culture with breast cancer cell lines alone or with M1 macrophages. Interestingly, within the T47D cell line an increase in mammosphere formation was observed with the co-culture of THP-1 monocytes (Figure 6A/6B). Co-culture of both ER− breast cancer cell lines with M2 macrophages also increased mammosphere formation in comparison to breast cancer cell lines co-cultured with M1 macrophages. However, unlike the ER+ cell lines, co-culture with all other cells types (THP1- monocytes, macrophages and M1 macrophages) resulted in a reduction in mammosphere formation (Figure 6C/6D).

### Zoledronate targets the macrophage microenvironment to reduce breast cancer cell migration and mammosphere formation

**Figure 7:**
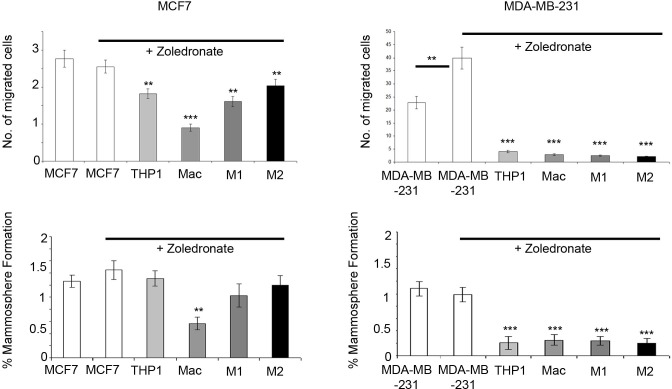
Targeting macrophages with Zoledronate reduces breast cancer cell migration and mammosphere formation Breast cancer cell lines (MCF7, T47D, MDA-MB-231 and MDA-MB-468) were treated with 1uM Zoledronate for 48hrs (white bars). Additionally, cells were treated with Zoledronate in co-culture with THP1-monocytes, macrophages or M1/M2 macrophages and compared to breast cancer cells alone. Mammosphere formation and migration of breast cancer cells was assessed following treatment and co-culture. Bar charts represent the mean % of cells; ±SEM. *P* values were generated using a two sided t-test assuming equal variance. Comparisons made were between untreated breast cancer cells and Zoledronate treated breast cancer cells, and comparison between breast cancer treated cells (alone) and treated cells in co-cultured with THP-1, macrophages and M1 and M2 macrophages * indicates significance, *p* < 0.05. **p < 0.01 ***p < 0.001.

Having shown the effects of monocytes and macrophages on breast cancer cell migration and mammosphere formation, we used the *in vitro* co-culture system as a model to test the effects of the bisphosphonate Zoledronate, both on breast cancer cells alone and within the co-culture setting. Zoledronate treatment of MCF7 and MDA-MB-231 cells in homotypic culture caused no reduction in migration or mammosphere formation. In fact, a small increase was observed in mammosphere formation in MDA-MB-231 cells. However, Zoledronate treatment of breast cancer cells whilst in co-culture with macrophages significantly reduced breast cancer migration and mammosphere formation (Figure 7).

## DISCUSSION

Macrophages are a major component of the breast cancer microenvironment. In this study, we have identified increased macrophage infiltration within Luminal B and ERBB2 subtypes, and demonstrated that macrophage infiltration is associated with tumour recurrence, high grade and positive lymph node status which is consistent with previous published literature [16-19]. Using *in vitro* models we have also successfully identified a role for macrophages and monocytes in breast cancer, dependent upon breast cancer subtype, and have shown how macrophages exert pro-tumourigenic effects through increasing migration and mammosphere formation, a marker of stem-like cell activity.

We used the THP-1 monocyte cell line as a model for macrophage differentiation and polarisation and to model the *in vivo* microenvironment of solid breast tumours. THP-1 cells have been widely used in macrophage research and have been shown to closely model primary macrophages extracted from whole blood [29-31]. Subsequently we polarized macrophages into distinct subtypes using LPS/IFN or IL4/IL13 to establish M1 and M2 subtypes respectively [32, 33]. A number of molecular markers have been identified to distinguish between macrophage subtypes, within this study we used ROS and HLA-DR which showed significant differences and confirmed adequate polarization between M1 and M2 subtypes respectively.

**Figure 8:**
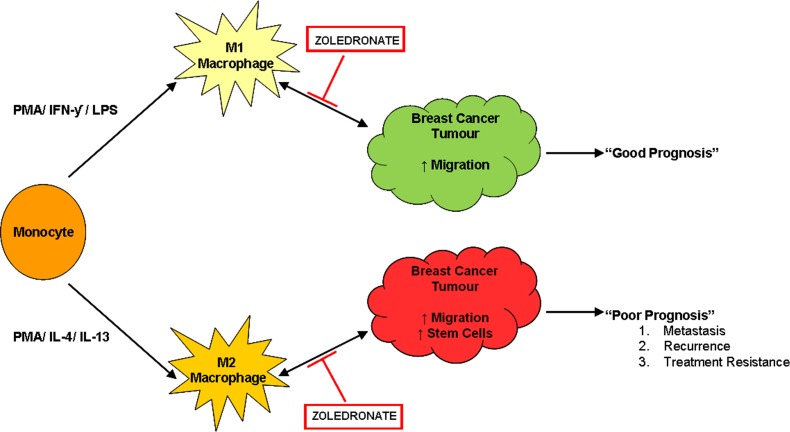
Zoledronate targets the pro-tumourigenic macrophage microenvironment Monocytes can be polarized into two distinct types of macrophages, M1 and M2, using phorbol 12-myristate 13-acetate (PMA), interferon gamma (IFN-γ) and lipopolysaccharide (LPS) or PMA and interleukin 4 (IL-4) and interleukin 13 (IL-13) respectively. M1 macrophages, classically referred to as “pro-inflammatory macrophages” are known to secrete many factors such as tumour necrosis factor alpha (TNF-α) and reactive oxygen species (ROS) and likely unknown factors. Within our model M1 macrophages stimulate breast cancer migration. M2 macrophages classically referred to as “pro-tumourigenic macrophages” are known to secrete numerous factors including matrix metalloproteinase's (MMPs) and epidermal growth factor (EGF). Within our model M2 macrophages stimulate breast cancer migration and stem cell-like activity. The interaction between macrophages and cancer cells is bi-directional whereby cancer cells also secrete proteins, for example colony stimulating factor (CSF) which recruits M2 macrophages to the tumour. Zoledronate inhibits the effects that both M1 and M2 macrophages exert upon breast cancer cells, with no direct effect on the breast cancer cells in the absence of a macrophage microenvironment. The mechanism of action of Zoledronate is therefore likely to effect the interaction between macrophages and breast cancer cells.

Having established the differentiation and polarization of macrophages, co-culture experiments were performed using a panel representing ER+ and ER− breast cancer cell lines to assess key cancer cell characteristics. ER+ breast cancer migration was increased with co-culture of macrophages, whilst in ER− breast cancer migration was inhibited. Despite this, the presence of macrophages significantly increased migration compared to monocytes. M2 macrophages significantly increased mammosphere formation across all breast cancer cell lines. This suggest that the specialised M2 macrophages, unlike M1 macrophages, secrete a specific set of proteins that directly regulate stem cell activity, and support the idea that tissue associated macrophages, which contribute to poor prognosis, are of an M2 phenotype. Identification of these specific “stem-like cell promoting” proteins will aid in developing novel drugs to treat the pro-tumourigenic microenvironment. Using our model we have identified a potential treatment to overcome the pro-tumourigenic effects of the macrophage microenvironment. Treatment of cancer cells with Zoledronate, only when in co-culture, reduced migration consistent with a recent publication [27]. In addition we identified novel effects, whereby Zoledronate reduced breast cancer mammosphere formation when in co-culture represented schematically in Figure 8. Zoledronate is a nitrogen-containing bisphosphonate, Zoledronate (zoledronic acid) initially used in the treatment of osteoporosis and more recently in advanced breast cancer due to its positive actions within bone. However it was observed that Zoledronate may also inhibit breast cancer progression and invasion [26]. *In vitro* and *in vivo* studies have shown anti-tumourigenic effects on breast cancer tumours, including the prevention of metastasis into the extracellular matrix however its exact mechanism of action is unclear [34, 35]. Growing evidence suggests that Zoledronate may not act directly on cancer cells, but within the microenvironment, potentially by reprogramming macrophages into a non-tumourigenic phenotype. *In vivo* murine models treated with Zoledronate showed impaired M2 macrophage recruitment and a switch from M2 to M1 macrophage phenotype [34, 36, 37]. This may be as a result of reduced IL13 expression, a key cytokine for the activation of M2 macrophages [38, 39]. The latter suggests that Zoledronate may have specific effects via M2 pro-tumourigenic macrophages rather than directly on breast cancer cells [27]. This is consistent with our results and would suggest that Zoledronate acts upon M2 macrophages to re-polarize cells into an M1 phenotype. In turn, this would prevent migration and mammosphere activity, both essential characteristics leading to tumour progression and metastasis, and could explain the decreased survival of patients correlating to macrophage infiltration. The mechanism of macrophage reprogramming exerted by Zoledronate is yet to be discovered. Despite this, Zoledronate is an exciting new treatment for breast cancer patients that can be used in both a primary and metastatic setting, to overcome the powerful pro-tumourigenic effects of the microenvironment.

In conclusion, macrophages are a key component of the breast cancer microenvironment which when present, in particular the M2 subtype, predict poor prognosis, through the promotion of migration and stem cell activity. Macrophages therefore represent a new therapeutic target for the treatment of breast cancer. Zoledronate exerts anti-tumourigenic effects by targeting the macrophage microenvironment to reduce cancer cell migration and stem cell activity. Additional clinical trials, in a neoadjuvant and metastatic setting are required, with increased dosing regimes, to fully assess the potential clinical benefit of Zoledronate in breast cancer treatment.

## MATERIALS AND METHODS

### Cell lines

All cell lines were purchased from ATCC; MDA-MB-231, MDA-MB-468 (ER negative) MCF7, and T47D (ER positive) and THP-1 (monocytes). Cell lines were authenticated by multiplex PCR assay using the AmpFlSTR Indentifiler PCR amplification kit (Applied Biosystems, Life Technologies Corporation, #4322288) and confirmed as mycoplasma free. THP-1, MDA-MB-231 and MDA-MB-468 were cultured in RPMI complete medium (RPMI/10% FCS/1% Sodium pyruvate/2mM L-glutamine/PenStrep) whilst MCF7 and T47D were grown in DMEM complete medium (DMEM/10% FCS/2mM L-glutamine/PenStrep). Cells were maintained in a humidified incubator at 37°C at an atmospheric pressure of 5% (v/v) carbon dioxide/air.

### Monocyte-macrophage differentiation

THP-1 monocytes were plated (200,000 cells/ml) in RPMI supplemented with 200nM Phorbol myristate acetate (PMA) for 72 hrs to achieve macrophage differentiation. Media was then supplemented with either 100ng/ml IFN-ƴ and 100 ng/ml LPS (M1 macrophage subtype) or 20 ng/ml IL4 and 20 ng/ml IL13 (M2 macrophage subtype) and cultured for a further 5 days prior to harvesting, to achieve polarization into macrophage subgroups.

### Imaging

Imaging of THP-1 monocytes/macrophages was carried out using an Olympus CKX41SF2-5 microscope, 10X at magnification. For fluorescence imaging, an X-cite 120 fluorescence camera and illumination system was used. Images were taken after the 8 day incubation to induce macrophage differentiation.

### Monocyte-macrophage flow cytometry analysis

Following monocyte-macrophage differentiation and polarization 400,000 cells were incubated with 4ul CD14-PE, CD11b-PE, HLA-DR-PE or 5 μM CM-H_2_DCFDA in the dark for 10 minutes at 37ºC. Cells were resuspended in 500ul PBS and fluorescence analyzed by FACS. Cells were analysed using the Becton Dickinson FACS Calibur and data was processed using BD CellQuest™ Pro V6.0 and analysed using WinMDI V2.8 software.

### Lysotracker

Untreated THP-1 monocytes or THP-1 cells treated with PMA, were incubated at a density of 1 x10^6^ cells/ml with lysotracker reagent at 37ºC for 1 hour, followed by fixation with 4% formalin for 15 minutes and mounted with Prolong DAPI.

### *In vitro* co-culture model

Breast cancer cells (6 × 10^5^) were co-cultured with either THP-1 monocytes, macrophages, M1 macrophages or M2 macrophages (6 × 10^5^), or breast cancer cells alone in 6-well 24 mm Transwell® sterile plates, separated by a 0.4 μm pore polycarbonate membrane insert (Corning, catalogue #3412). Cells were incubated for 48 hours at 37°C before harvesting for subsequent analysis of breast cancer cell lines.

### Mammosphere culture

Following co-culture, breast cancer cells were collected and a single cell suspension prepared using enzymatic (1x Trypsin-EDTA, Sigma Aldrich, #T3924), and manual disaggregation (25 gauge needle). Cells were plated at a density of 500 cells/cm^2^ in non-adherent conditions in mammosphere medium (DMEM-F12/B27/20ng/ml EGF/PenStrep). Cells were grown for 5 days and maintained in a humidified incubator at 37°C at an atmospheric pressure in 5% (v/v) carbon dioxide/air. Mammospheres > 50μm were counted using an eye piece graticule.

### Migration assay

Following co-culture, breast cancer cells were collected and migration assessed using transwell chambers with a diameter of 6.5 mm and a pore size of 8 μm (Corning, Inc. #3422). Cells (50,000) were resuspended in 150 μl serum-free media and added to the upper migration chamber. Cells were allowed to migrate for 5 hours (MDA-MB-231) or overnight (MCF7, T47D, MDA-MB-468) towards media containing 10% FCS. Non-migrated cells were removed using a cotton swab. Migrated cells situated on the lower side of membranes were stained for 10 minutes with crystal violet solution (1% v/v crystal violet, 70% ethanol). Cells were viewed and counted using an Olympus CKX41SF2-5 microscope at X20 magnification (cells in 15 fields were counted). Experiments were repeated in triplicate. The percentage of cells that had migrated was calculated.

### Cell growth assay

Following co-culture breast cancer cells were plated in a 96-well tissue-culture plates at a ratio 2500 cells/100 μl (MDA-MB-231) or 5000 cells/100 μl (MDA-MB-468, MCF7, T47D). Cells were incubated for 24 hours at 37°C, the media was removed and 20 μl alamarBlue® (AB)/complete media (1:20) was added to each well. The pate was re-incubated for 1 hour at 37°C. Fluorescence was measured at an excitation wavelength of 544 nm and an emission wavelength of 590 nm on a FLUOstar Omega software V1.20 (BMG Labtech). After measurement the AB/complete media solution was removed and the plate washed twice with PBS. Complete media (100 μl/well) was added for a further 24 hours and the process repeated.

### Cell apoptosis/necrosis

Following co-culture, 400,000 breast cancer cells were re-suspended in 500 μl 1X Annexin V binding buffer with 5 μl of Annexin V-FITC (Apoptosis) and 5 μl Propidium Iodide (Necrosis). Cells were incubated in the dark for 15 minutes at room temperature. Cells were then analysed by FACS. Cells were analysed using the Becton Dickinson FACS Calibur and data was processed using BD CellQuest™ Pro V6.0 and analysed using WinMDI V2.8 software.

### Zoledronate (ZOL) treatment

Breast cancer cells were co-cultured with or without THP-1 monocytes, macrophages, M1 and M2 subtypes as previously described and treated with or without 1μM ZOL (Zoledronic acid, Novartis) and incubated for 48 hours at 37°C. Breast cancer cells were collected and migration and mammosphere formation assayed.

### Primary breast cancer microarray

Affymetrix gene expression data representing a total of 1107 primary breast tumours from six previously published microarray studies [40-45] were integrated as described previously using ComBat [46] to remove batch effects [28]. Centroid prediction [47] was used to assign the tumours from each dataset to the five Norway/Stanford subtypes (Basal, Luminal A, Luminal B, ERBB2 and Normal-like [48]. Histological grade information was taken from the original datasets.

### Primary breast cancer tissue microarray

The breast cancer samples were obtained from 144 patients undergoing surgical resection at Malmo University Hospital, Sweden, between 2001 and 2002 and all patients received adjuvant chemotherapy. Additional clinical information has been previously described [49]. The study was approved by the regional ethical committee in Lund, Sweden.

FFPE TMA sections were deparaffinised, rehydrated and antigen retrieval was performed using Dako target retrieval solution (Dako). For IHC, anti-CD68 antibody was used (M0814. DAKO) at a dilution of 1 in 1500. Isotype control was performed to ensure antibody specificity. The number of CD68-positive cells within each TMA core was counted at x400 magnification and patients subsequently divided into subgroups by the mean into low and high expression.

### Statistical methods

Throughout the paper data is represented as mean ±SEM taken over a minimum of three independent experiments. Statistical significance was measured using parametric testing, assuming equal variance, with standard t-Tests for two paired samples used to assess difference between test and control samples, unless otherwise stated.

## SUPPLEMENTARY MATERIALS FIGURES


