# Supplementary material for: Monocytes and macrophages, implications for breast cancer migration and stem cell-like activity and treatment
Source: Oncotarget. 2015 May 19;6(16):14687–99. doi: 10.18632/oncotarget.4189 (PMC4546497; doi:10.18632/oncotarget.4189)
Supplement: Supplementary file 1 [file oncotarget-06-14687-s001.pdf]

# Monocytes and macrophages, implications for breast cancer migration and stem cell-like activity and treatment

**Supplementary Material**

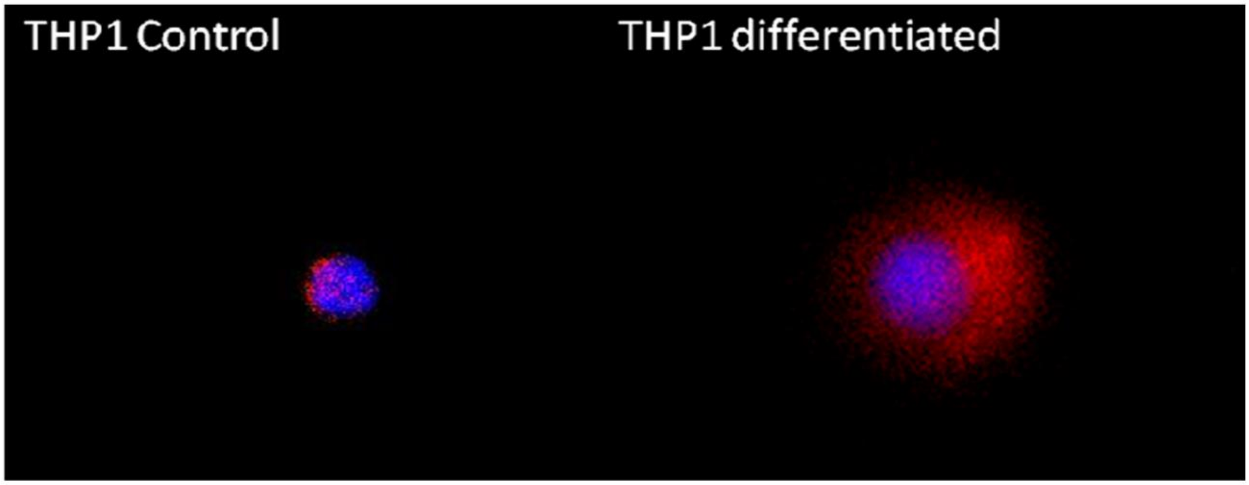


**Supplementary Figure 1: Increased lysosomes confirm monocyte-macrophage differentiation.** THP-1 monocytes either (A) untreated or (B) treated with PMA were labelled with Lysotracker (Red) and Prolong Dapi (Blue)

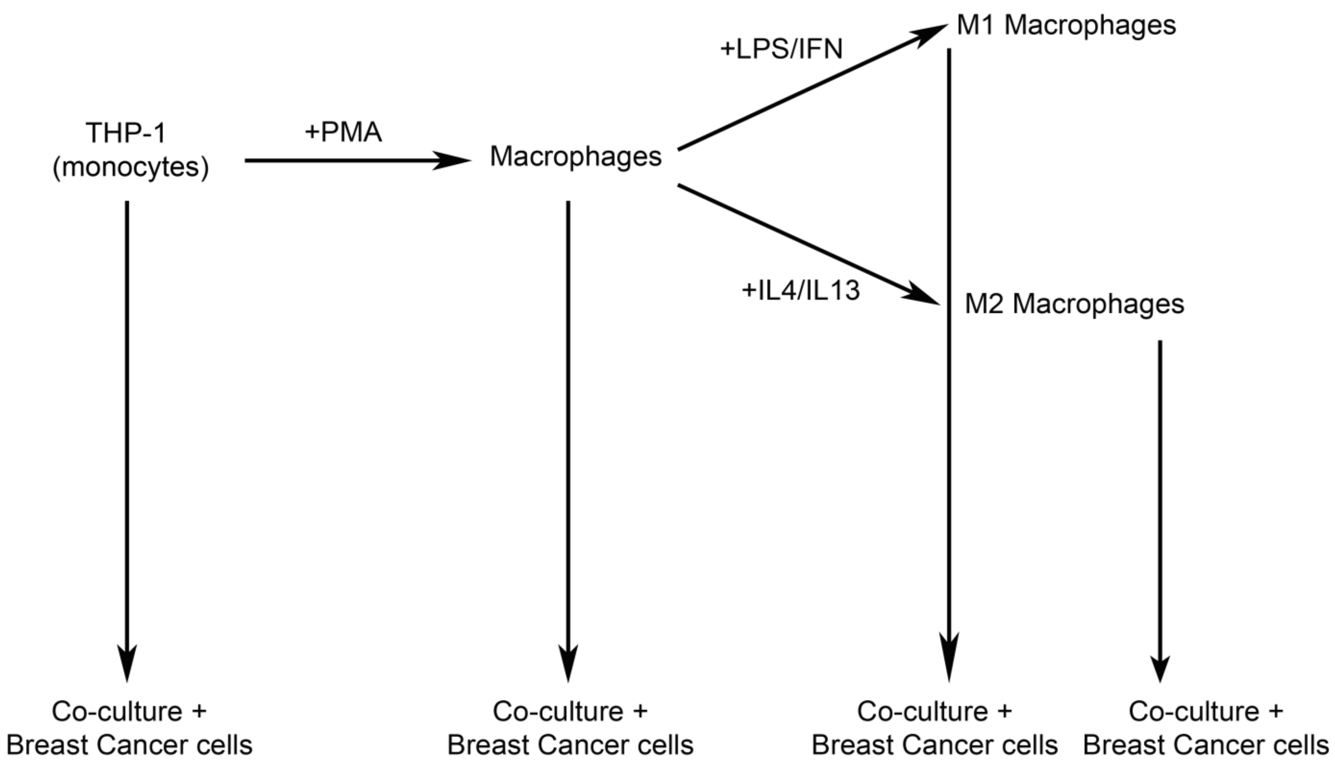


**Supplementary Figure 2: Diagrammatic representation of monocyte-macrophage model.** THP-1 monocytes were treated with PMA to induce macrophage differentiation. Cells were further treated with IFN /LPS (M1) or IL4/IL13 (M2) to achieve macrophage polarization. THP-1 cells from each stage of differentiation and polarization were co-cultured with breast cancer cell lines (MCF7, T47D, MDA-MB-231 and MDA-MB-468) for 48hrs. Breast cancer migration, mammosphere formation, proliferation and apoptosis/necrosis were then assessed.
